# The spectrum of unhealthy drug use and quality of care for hypertension and diabetes

**DOI:** 10.1186/1940-0640-10-S1-A27

**Published:** 2015-02-20

**Authors:** Theresa W Kim, Jeffrey H Samet, Debbie M Cheng, Judith Bernstein, Na Wang, Jacqueline German, Richard Saitz

**Affiliations:** 1 Clinical Addiction Research and Education Unit, Section of General Internal Medicine, Boston Medical Center, Boston University School of Medicine, Boston, MA, 02118, USA; 2 Department of Community Health Sciences, Boston University School of Public Health, Boston, MA, 02118, USA; 3 Department of Biostatistics, Boston University School of Public Health, Boston, MA, 02118, USA; 4 Data Coordinating Center, Boston University School of Public Health, Boston, MA, 02118, USA

## Background

Addiction is associated with medical consequences, due to direct drug effects, poor adherence to health-care recommendations, and poor quality of care. But little is known about how the spectrum of illicit drug use in primary care patients affects quality of medical care measures for chronic conditions. The objectives of this study were to determine the association between drug use (drug, frequency of use, and severity) and quality measures indicating failure to meet criteria for good blood pressure (BP) and blood glucose (BG) control among primary care patients identified by screening as using illicit drugs who also have hypertension and/or diabetes.

## Materials and methods

The study population was adult patients presenting for primary care visits in an urban, safety-net hospital with recent illicit drug use or prescription drug misuse identified by screening and either hypertension or diabetes mellitus. The outcomes were failure to control: a blood pressure (primary), defined as systolic BP of 140 or higher or diastolic BP of 90 or higher, and b) blood glucose (secondary), defined as hemoglobin A1c of 8 percent or higher (National Committee for Quality Assurance (NCQA) and Healthcare Effectiveness Data and Information Set [HEDIS] measures). Main independent variables were: days of drug use in the past month; drug type (i.e., opioid, cocaine, marijuana); and severity of use (Alcohol, Smoking and Substance Involvement Screening Test global drug risk score (tertiles). We fit separate longitudinal logistic regression models for each measure of drug use and each outcome.

## Results

Overall, 40 percent (66/164) of the sample with hypertension failed to meet criteria for BP control and 42 percent (26/62) of those with diabetes failed to meet criteria for BG control. No significant associations were detected for any measure of drug use and BP or BG control, except those reporting cocaine use had higher odds of failing to meet criteria for BG control compared to those reporting marijuna use (adjusted odds ratio (AOR) 8.8, 95% CI: 1.9, 41.9). Higher severity of drug use was associated with higher odds of failing to meet criteria for BG control in an unadjusted model (OR 3.1, 95%CI 1.0, 9.2); however, this association was not significant after controlling for age, gender, and race/ethnicity.

## Conclusions

In this cohort of primary care patients with drug use, type of drug but not frequency of use or severity was significantly associated with failure to meet quality measure criteria for control of hypertension and diabetes.

## Trial registry

Trial registrationidentifying number NCT00876941

